# Escalation with Overdose Control is More Efficient and Safer than Accelerated Titration for Dose Finding

**DOI:** 10.3390/e17085288

**Published:** 2015-07-27

**Authors:** André Rogatko, Galen Cook-Wiens, Mourad Tighiouart, Steven Piantadosi

**Affiliations:** Biostatistics and Bioinformatics Research Center, Cedars-Sinai Medical Center, 8700 Beverly Blvd., PACT, Suite 900C, Los Angeles, CA 90048, USA

**Keywords:** dose finding, cancer early trials, phase I trials, escalation with overdose control, accelerated titration, EWOC

## Abstract

The standard 3 + 3 or “modified Fibonacci” up-and-down (MF-UD) method of dose escalation is by far the most used design in dose-finding cancer trials. However, MF-UD has always shown inferior performance when compared with its competitors regarding number of patients treated at optimal doses. A consequence of using less effective designs is that more patients are treated with doses outside the therapeutic window. In June 2012, the U S Food and Drug Administration (FDA) rejected the proposal to use Escalation with Overdose Control (EWOC), an established dose-finding method which has been extensively used in FDA-approved first in human trials and imposed a variation of the MF-UD, known as accelerated titration (AT) design. This event motivated us to perform an extensive simulation study comparing the operating characteristics of AT and EWOC. We show that the AT design has poor operating characteristics relative to three versions of EWOC under several practical scenarios. From the clinical investigator's perspective, lower bias and mean square error make EWOC designs preferable than AT designs without compromising safety. From a patient's perspective, uniformly higher proportion of patients receiving doses within an optimal range of the true MTD makes EWOC designs preferable than AT designs.

## 1. Introduction

Clinical trials of new anti-cancer therapies are widespread, critically important tools in the search for more effective cancer treatments. Cancer trials typically proceed through several distinct phases. The major objective in dose finding (phase I) trials is to identify a working-dose for subsequent studies, whereas the major endpoint in phase II and III trials is treatment efficacy. The dose sought is typically referred to as the maximum tolerated dose (MTD), and its definition depends on the treatment under investigation, the severity and reversibility of its side effects, and on clinical attributes of the target patient population. Specifically, the MTD, γ, is defined as the dose expected to produce some degree of medically unacceptable, dose-limiting toxicity (DLT) in a pre-specified proportion θ of patients [1]:

(1) P{DLT|Dose=γ}=θ

One of the very few options for patients with advanced cancer refractory to available treatment is to participate in dose-finding (phase I) oncology trials. Horstmann *et al*. [2] reviewed all non-pediatric phase I oncology trials sponsored by the Cancer Therapy Evaluation Program at the National Cancer Institute between 1991 and 2002. They analyzed 460 trials involving 11,935 participants, all of whom were assessed for toxicity and 10,402 of whom were assessed for a response to therapy. The overall response rate was 10.6%, with considerable variation among trials. These results demonstrate that it is reasonable to expect a therapeutic intent from a phase I trial. Thus, to increase the chances that a patient will benefit from participating in a phase I trial one should improve the design of these trials so that the number of patients receiving optimal doses is maximized. Consequently, more patients would be treated with therapeutic doses of promising new agents, and fewer patients would have to suffer the deleterious effects of toxic doses.

Since there is an ethical motivation to not harm the patient, controlling this risk is a desirable goal for physicians and patients. Escalation with Overdose Control (EWOC) was the first dose-finding procedure to directly incorporate the ethical constraint of minimizing the chance of treating patients at unacceptably high doses [3]. Its defining property is that the expected proportion of patients treated at doses above the MTD is equal to a specified value α, the feasibility bound. The method is flexible enough to allow prior information about the drug from laboratory or animal studies to be incorporated in the model, makes use of all the information available at the time of each dose assignment, controls the probability of overdosing patients at each stage, allows the estimation of the precision of the MTD, is optimally Bayesian feasible [4], produces a sequence of doses that converges in probability to the MTD [4], is coherent [5,6], and accounts for patients' pre-treatment characteristics [7–9]. EWOC can be implemented with the user friendly software EWOC [10,11] or WinBUGS [12] for general class of prior distributions [13], or ordinal toxicity grades [14]. EWOC allows flexible patient enrollment, and conforms to the ethical goal of maximizing the number of patients receiving optimal doses. At the time this article was written, we were aware of twenty peer-reviewed articles describing trials designed with EWOC.

In June 2012 a protocol accompanying the Investigational New Drug (IND) application “A Phase 1 Clinical and Pharmacokinetic Study of OVI-117 Administered Once Weekly for Three Consecutive Weeks, with Cycles Repeated Every Four Weeks, to Patients with Advanced Solid Tumors” designed with EWOC was submitted to the U.S. Food and Drug Administration (FDA) and was reviewed by the Office of Hematology and Oncology Products (OHOP), Center for Drug Evaluation and Research (CDER). The interaction led to the rejection of EWOC and the imposition of the accelerated titration (AT) design, which is a variation of the “modified Fibonacci” up-and-down (MF-UD) method of dose escalation.

The following “Clinical Deficiencies” related to the statistical design were identified by the FDA: (1) “Please modify your protocol to clearly state the amount of study drug that will be administered at each dose level.” (2) “It appears that you plan to treat 1 patient at each dose level. Please modify your protocol so that 1 patient will be treated at each dose level only until the first patient experiences a grade 2 adverse event that is at least possibly related to study drug. After that point, at least 3 patients should be treated at each dose level (including the level at which the grade 2 event occurred).” (3) “Please remember to include these conditions in the design: Do not increase dose by more than 100% at any time. Do not increase the dose by more than 50% after the first instance of grade 2 toxicity. Enroll enough patients at each dose level before escalating the dose to the next level. If ≥ 33% of patients at a dose level develop DLTs, do not escalate the dose even if the model recommends it.”

The fact that, in the past, the FDA approved several study protocols using EWOC with continuous support and with one patient per dose level (for example [15,16]) and that we provided detailed theoretical justifications supporting all aspects of the EWOC design with peer-reviewed publications were not sufficient for the proposed EWOC design to be accepted this time by the FDA and we were forced to adopt the AT design.

The standard 3 + 3 or MF-UD is by far the most used design in dose-finding cancer early trials [17]. MF-UD was the state-of-the-art in 1971 when it was first used in cancer clinical studies. Since then, over one hundred methodological papers proposing or evaluating dose escalation designs have been published and MF-UD has always shown inferior performance when compared with its competitors regarding maximizing number of patients treated at optimal doses and minimizing number of patients under or overdosed [17]. AT was first proposed by Storer [18] and popularized by Simon and colleagues [19].

The goal of this manuscript is to compare the operating characteristics regarding safety and efficiency between AT and EWOC designs through extensive simulations studies under several practical scenarios. A comparison between these two methods has never been performed before. An innovative aspect is the use of a continuous dose support, instead of a fixed set of doses to implement the AT design. This allowed comparison of different escalation rates in both phases of the AT design, and different starting doses. The use of several initial doses and escalation rates for the accelerated and 3 + 3 phases in the simulations produced 150 distinct dose levels and provide the rationale for adopting a continuous support for dose. Previous work by Lin and Shih [20] compared the operating characteristics of six up-and-down designs, including the AT, using five dose levels. Gerke and Siedentop [21] considered the AT design, two up-and-down designs, the maximum likelihood version of the continual reassessment method, and a Bayesian method that is implemented in the software Bayesian assisted decision-making in early phase trials (ADEPT) using 10 dose levels. The possibility of exploring a large number of dose levels and not assigning a priori one of them as the true MTD brings the simulated trials closer to what happens in real clinical trials, where there is no assurance that one of the pre-selected dose levels is the true MTD. In addition, five models that specify the dose-toxicities relationship were used in the simulation studies to generate responses.

## 2. Methods

### 2.1. Models, Responses and General Settings

Let *G* = 0, 1, …, 4 be the maximum grade of toxicity experienced by a patient by the end of one cycle of therapy and define DLT as a maximum of grade 3 or 4 toxicity. Let:

(2) $$Y=\{\begin{array}{c} 0\;\text{if}\;G=0\;\text{or}\;1\\ 1\;\text{if}\;G=2\\ 2\;\text{if}\;G=3\;\text{or}\;4 \end{array}$$

The dose-toxicities relationship is modeled by:

(3) $$P(Y\geq j|x)=F(\alpha_{j}+\beta x),\;j=1,2,$$

where *F*(·) is a known strictly increasing cumulative distribution function This implies that α_2_ < α_1_. We consider the case β_1_ = β_2_ = β so that the working Model (3) is the proportional odds model. Moreover, we assume that β > 0 so that the probability of DLT is an increasing function of dose. Doses are assumed to be continuous and standardized to the interval [0, 1]. The MTD, γ, is defined as the dose that is expected to produce DLT in a specified proportion θ of patients:

(4) $$P(Y=2|x=\gamma )=F(\alpha_{2}+\beta \gamma )=\theta$$

We reparameterize Model (3) in terms of ρ_0_, the probability that a DLT manifests within the first cycle of therapy for a patient given dose *x* = 0:

(5) $$\rho_{0}=P(Y=2|x=0),$$

ρ_1_, the probability that a grade 2 or more toxicity manifests within the first cycle of therapy for a patient given dose *x* = 0:

(6) $$\rho_{1}=P(Y\geq 1|x=0),$$

and the MTD γ. This reparameterization is convenient to clinicians since γ is the parameter of interest, and both ρ_0_ and ρ_1_ are easier to interpret in practice. It can be shown that:

(7) $$\begin{array}{c} \alpha_{1}=F^{-1}(\rho_{1}),\alpha_{2}=F^{-1}(\rho_{0}),\\ \beta =\frac{1}{\gamma }(F^{-1}(\theta )-F^{-1}(\rho_{0})). \end{array}$$

Five models that specify the dose-toxicities relationship in Equation (3) were used in the simulation studies to generate responses:

- Logistic with link function *F*(*w*) = 1/(1+*e ^−w^* ),
- Normal link function with shape parameter σ = 0.5,
- Normal link function with shape parameter with σ = 2,
- Non-proportional odds model β_1_≠β_2_) with logistic link function used on (A) with ρ_0_ = 0.126, and
- Non-proportional odds model with logistic link function used on (A) with ρ_0_ = 0.02.

**Figure 1:**
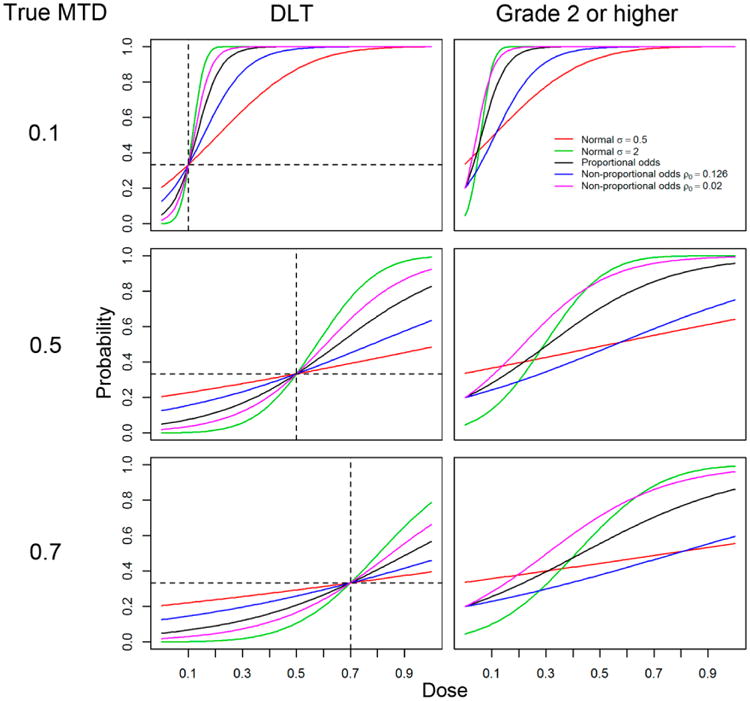
Dose-toxicity relationship for *P*(DLT|dose) on the left hand side and for *P*(grade 2 or higher|dose) on the right hand side, for three selected values of true MTD, for five models considered in the simulation studies: proportional odds logistic (**black**), normal with σ = 0.5 (**red**), normal with σ = 2 (**green**), non-proportional odds with ρ_0_ = 0.126 (**blue**), and non-proportional odds with ρ_0_ = 0.02 (**magenta**). The horizontal dashed lines represent the target probability of DLT θ = 0.33 and the vertical lines correspond to the true values of the MTDγ.

Figure 1 shows the dose-toxicity relationship for *P*(DLT|dose) where the target probability of DLT is set to θ = 0.33 on the left hand side and for *P*(grade 2 or higher|dose) on the right hand side, for three selected values of true MTD, and for five models considered in the simulation studies. The ρ_0_ values used for testing robustness with non-proportional odds are the same as those from [14]. The non-proportional odds logistic model is a logistic link function of the same form that the proportional odds logistic uses, but with parameters chosen to perturb the response function so that it passes through the point (γ, θ), as in the main simulation results, but with a different intercept (0, ρ_0_). Three selected values for the true MTD: {0.1, 0.5, 0.7} combined with three chosen values for the true ρ_1_: {0.2, 0.5, 0.8} produced nine scenarios used in the simulations. One thousand trials were simulated for each scenario. The designs compared in the study are described next.

### 2.2. AT Designs

Doses in the AT designs were incremented based on a percentage of the previous dose [19]. Two starting doses were selected: 0.01 and 0.1. The AT design comprises two phases. In the first phase (accelerated phase), only one patient is treated at each dose level and doses increase at a faster rate than in the second phase. The second phase enrolls three patients and dose escalation proceeds using the standard MF-UD algorithm. Three sets of dose increments were chosen:

1. Double the dose in the accelerated phase and increase by 50% in the MF-UD phase (recommended by FDA);
2. Increase by 69% in the accelerated phase and 30% in the MF-UD phase; and
3. Increase by 96% in the accelerated phase and 40% in the MF-UD phase.

Six versions of AT design were studied by combining two starting doses and three sets of dose increments. Thus, each version is identified by a vector with three elements:

(8) (starting dose,accelerated phase increase rate,MF-UD increase rate)

resulting in: (0.01, 2, 1.5), (0.1, 2, 1.5), (0.01, 1.69, 1.3), (0.1, 1.69, 1.3), (0.01, 1.96, 1.4), and (0.1, 1.96, 1.4).

Each trial starts in the accelerated phase, and the first patient at the starting dose is given a safe response (grade 0 or 1). The algorithm used in the simulations for the AT designs proceeds as follows:

Start trial in the accelerated phase by treating patients at the initial dose. Denote the dose level being used to treat patients as the current dose level.
Accrue and treat one patient at the current dose level.
Check the toxicity in the one patient at the current dose level.
  3a. If there is a grade 2 toxicity or higher, end the accelerated phase and go to 5.
  3b. If there is no toxicity, escalate the dose and go to 4.
4. Escalate if possible.
  4a. If the current dose level is the highest dose level; stop the trial and declare that the MTD is higher than the highest dose level.
  4b. Otherwise, escalate to the next-higher dose level; go to 2. The next higher dose level is the current dose level multiplied by the increment used in the accelerated phase (e.g., 1.96).
Accrue and treat patients in the MF-UD design phase so that there are three to be treated at the current dose level.
  5a. If the maximum number of patients has been accrued, stop the trial. (The simulated trials were capped at 62 patients.)
Check the number of patients at the current dose level.
  6a. If there are three patients, go to 7.
  6b. If there are more than three patients, go to 8.
Check the number of toxicities (among three patients) at the current dose level.
  7a. If there are zero toxicities, escalate and go to 10.
  7b. If there is one toxicity, stay at the current dose and go to 5.
  7c. If there are two or three toxicities, declare that the MTD has been exceeded and go to 9.
Check the number of toxicities (among more than three patients) at the current dose level.
  8a. If there are zero toxicities, stop the trial and declare that the MTD is the current dose.
  8b. If there is one toxicity, escalate and go to 10 unless the MTD has been exceeded, then stop the trial and declare that the MTD is the current dose.
  8c. If there are two toxicities, stop the trial; the MTD is the current dose.
  8d. If there are three or four toxicities, declare that the MTD has been exceeded and go to 9 (it is impossible to have five or six toxicities among six patients at the same dose level under this MF-UD design).
The MTD has been exceeded.
  9a. If the current dose is the lowest dose, stop the trial; declare that the MTD is lower than the lowest dose level.
  9b. If the next-lower dose level has more than three patients, stop the trial and declare that the MTD is the next lower dose level; otherwise, set the current dose level to be the next-lower dose level and go to 5. The next lower dose level is the current dose level divided by the increment used in the MF-UD phase (e.g., 1.4)
Escalate if possible.
  10a. If the current dose level is the highest dose level; stop the trial and declare that the MTD is higher than the highest dose level.
  10b. Otherwise, escalate to the next-higher dose level; go to 5. The next higher dose level is the current dose level multiplied by the increment used in the MF-UD phase (e.g. 1.4)

### 2.3. EWOC Designs

Two designs denoted as *EWOC* and *EWOC-PO* were used. *EWOC* follows a binary logistic model and calculates the recommended dose for the next patient based on the occurrence of DLT (toxicity grades 3 or 4) or no DLT (toxicity grades 0, 1, or 2) response from previous patients [5]. *EWOC-PO* uses a proportional odds model assumption of dose-toxicity relationship and allows information on grade 2 toxicities to be incorporated as well [14]. For *EWOC* and *EWOC-PO*, the targeted probability of DLT was set to θ = 0.33, the feasibility bound α was fixed at 0.25 for every trial, and the sample size for each trial was fixed at 30.

We designed an MCMC sampler based on the Metropolis-Hastings algorithm [22,23] to implement *EWOC* and *EWOC-PO* designs. We also used WinBUGS [12] to estimate features of the posterior distribution of the MTD. WinBUGS code for *EWOC* was described in [13] and for *EWOC-PO* in [14]. Number of burn-ins and sample were both 4000. Convergence diagnostic was performed with boa package [24]. Vague priors for the model parameters were used:

(9) $$\begin{array}{c} \gamma \sim \text{Unif}[0,1]\\ \rho_{0}\sim \text{Unif}[0,\theta ]\\ \rho_{1}|\rho_{0}\sim \text{Unif}[\rho_{0},1]. \end{array}$$

Dose levels in the trial are selected in the interval [0, 1]. The adaptive design proceeds as follows. The first patient receives a dose *x*_1_ = 0 and a safe response (grade 0 or 1). Denote by Π*_k_*(γ) = Π(γ|*D_k_*) the marginal posterior c.d.f. of the MTD, *k* = 1, …, *n* − 1. The (*k* + 1)-st patient receives the dose $x_{k+1}=\prod_{k}^{-1}(\alpha )$ so that the posterior probability of exceeding the MTD is equal to the feasibility bound α = 0.25. This is the overdose protection property of EWOC, where at each stage of the design, one seeks a dose to allocate to the next patient while controlling the posterior probability of exposing patients to toxic dose levels. The trial proceeds until the planned number of patients are enrolled to the trial. At the end of the trial, we estimate the MTD as $\hat{\gamma }=\prod_{n}^{-1}(\alpha )$.

As mentioned in the Introduction, this simulation study was motivated by a real dose finding trial detailed in the protocol “A Phase 1 Clinical and Pharmacokinetic Study of OVI-117 Administered Once Weekly for Three Consecutive Weeks, with Cycles Repeated Every Four Weeks, to Patients with Advanced Solid Tumors” whose objective was to estimate the MTD of OVI-117 administered IV once weekly for three consecutive weeks on a 4-week cycle to patients with advanced malignancies. The MTD was defined to be the dose level of OVI-117 that when administered to a patient according to the schedule above results in a probability equal to θ = 0.33 that a dose limiting toxicity will be manifest within cycle 1. The dose selected for every patient in the trial was between the minimum dose 130 mg/m^2^ and the maximum allowable dose 3500 mg/m^2^ and was considered as a continuous quantity. The sample size was set to 30 patients. The dose escalation would have followed the EWOC method.

### 2.4. Efficiency and Safety Comparisons

Simulated trials following AT, *EWOC* and *EWOC-PO* designs were compared according to the average bias:

(10) $$\text{bias}=M^{-1}(\sum_{i=1}^{M}{\hat{\gamma }}_{i}-\gamma_{\text{true}}),$$

and the square root of the estimated mean square error:

(11) $$\text{MSE}=M^{-1}(\sum_{i=1}^{M}{\left({\hat{\gamma }}_{i}-\gamma_{\text{true}}\right)}^{2}),$$

where γ̂ *_i_* is the estimate of the MTD at the end of the *i*-th trial and *M* is the total number of simulated trials. In addition, models were compared with respect to the average proportion of patients exhibiting DLT, the proportion of trials for which the DLT rate exceeds θ + 0.05 = 0.38, the proportion of trials with estimated MTD within γ − 0.15γ and γ + 0.15γ (referred to as optimal dose), and the proportion of patients receiving optimal doses. It is important to highlight that, from the perspective of a patient participating in a dose finding trial, the best design is the one with the highest proportion of patients receiving optimal doses.

## 3. Results

**Figure 2:**
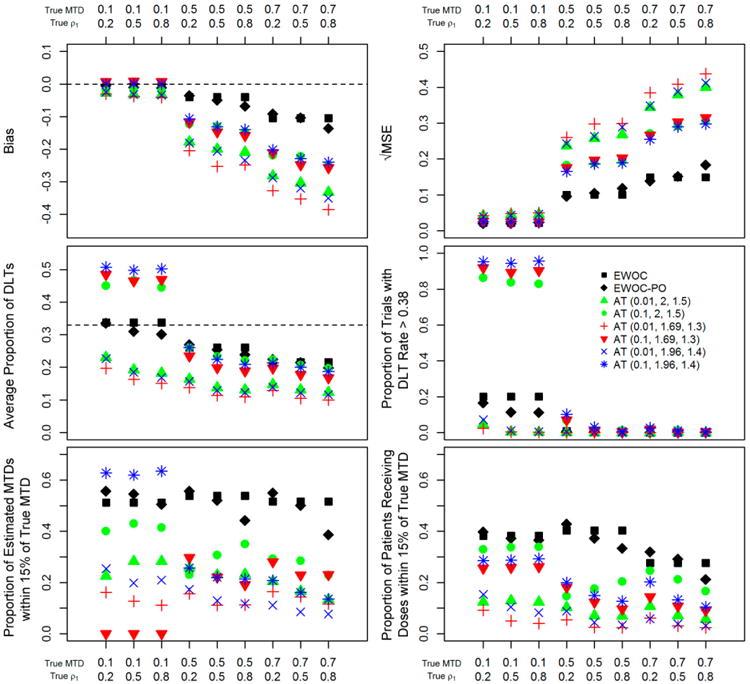
Summary statistics for trial efficiency and safety for *EWOC*, *EWOC-PO* and AT (starting dose, accelerated phase increase rate, MF-UD increase rate) under nine scenarios. Each symbol represents 1000 simulated trials.

**Figure 3:**
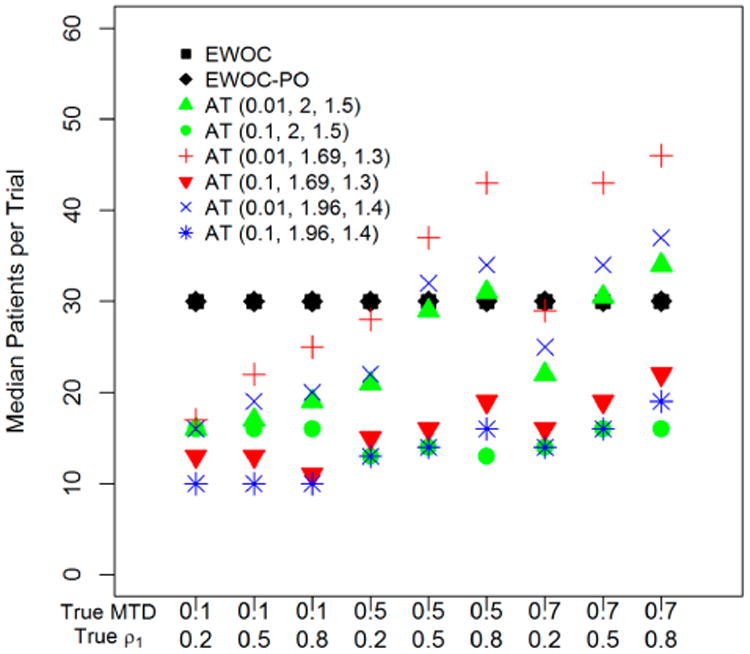
Median of the number of patients per trial for *EWOC*, *EWOC-PO* and AT (starting dose, accelerated phase increase rate, MF-UD increase rate) under nine scenarios. Each symbol represents 1000 simulated trials. The number of patients per *EWOC* and *EWOC-PO* trial is set to 30. The maximum number of patients per AT trial is 62 patients.

Figures 2 and 3 show results of eight types of designs (*EWOC, EWOC-PO*, and six versions of AT) simulated under nine scenarios of true MTD and true ρ_1_. Responses were simulated according to the proportional odds logistic model (model A, Section 2.1). Responses for *EWOC* were generated in the same way as for *EWOC-PO* and AT, however the grade 2 toxicities were considered as the same level of response as the lower grade toxicities, leaving two response levels for DLT or no DLT. It is worth noting that, for these Figures, responses were simulated with the same link function assumed by *EWOC* and *EWOC-PO* designs.

Regarding bias, all designs perform well when the true MTD = 0.1. On the other hand, when the true MTD > 0.1, AT designs display higher bias than *EWOC* and *EWOC-PO*. Similarly, √MSE is small for all designs when the true MTD = 0.1, while AT designs display higher √MSE than *EWOC* and *EWOC-PO* when the true MTD > 0.1. Regarding the average proportion of patients exhibiting DLT, *EWOC* and *EWOC-PO* are uniformly safe, being on target when the true MTD = 0.1 while AT designs tend to be mostly safe, except when the true MTD = 0.1 for three versions with starting dose = 0.1; in these instances the average proportion of patients exhibiting DLT nears 50%. Similar safety concerns are shown regarding the proportion of trials with DLT rate > 0.38 for three AT versions with starting dose = 0.1 when the true MTD = 0.1. Regarding the proportion of trials with estimated MTD around the optimal dose, except for AT (0.1, 1.96, 1.4) when the true MTD = 0.1, *EWOC* and *EWOC-PO* show higher proportions than AT. It is worth noting that AT (0.1, 1.96, 1.4) had poor safety characteristics. Regarding the proportion of patients receiving optimal doses, *EWOC* and *EWOC-PO* uniformly display higher proportions than the AT designs.

**Figure 4:**
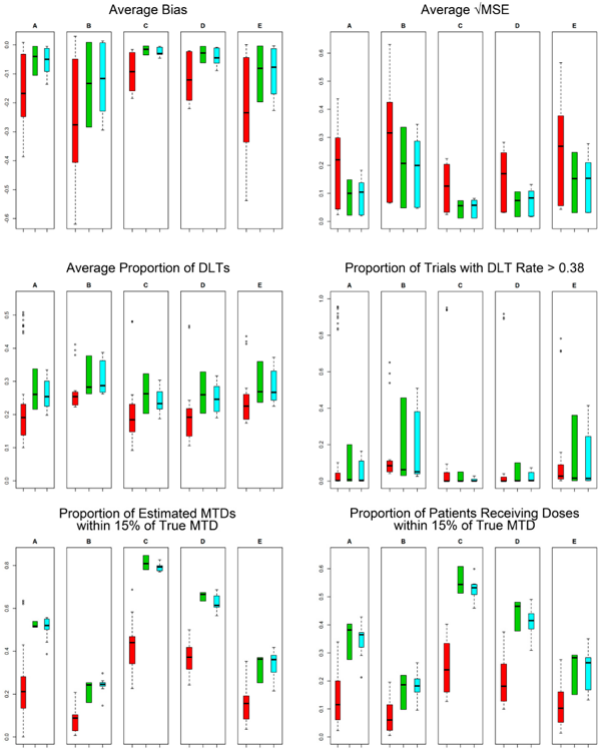
Summary statistics for trial efficiency and safety for AT (**red**), *EWOC* (**green**), and *EWOC-PO* (**blue**) designs for different models: (**A**) proportional odds logistic; (**B**) normal with σ = 0.5; (**C**) normal distribution with σ = 2; (**D**) non-proportional odds with ρ_0_ = 0.02; and (**E**) non-proportional odds with ρ_0_ = 0.126.

Figure 3 shows the median of the number of patients per trial for *EWOC*, *EWOC-PO* and AT according to nine scenarios. While the number of patients per *EWOC* and *EWOC-PO* trial was set to 30, the number of patients per AT trial varied considerably. Considering all AT simulated trials, the overall minimum 5th percentile was 5 patients and the overall maximum 95th percentile was 52. Figure 4 shows results of AT, *EWOC* and *EWOC-PO* designs for different models: (A) proportional odds logistic, (B) normal with σ = 0.5, (C) normal distribution with σ = 2, (D) non-proportional odds with ρ_0_ = 0.02, and (E) non-proportional odds with ρ_0_ = 0.126. In these series, the simulation results of two AT versions: (0.01, 2, 1.5) and (0.1, 2, 1.5) were combined for B, C, D, and E, whereas A combined all six AT versions. Also combined were the results of the nine simulation scenarios for the true MTD and true ρ1 by summarizing 1000 trials for each situation and plotting the nine summaries. Therefore, AT summarizes the results of 18,000 trials for B, C, D, and E, and 54,000 trials for A, while *EWOC* and *EWOC-PO* condenses the results of 9000 trials each. As in the previous simulations, *EWOC* and *EWOC-PO* designs assume a proportional odds model for the dose-toxicity relationship. Since responses simulated for models B, C, D, and E were generated by different distributions than those assumed by *EWOC* and *EWOC-PO*, they allow us to assess model robustness.

The different models had an effect on the performance of all of the designs. The normal distribution with σ = 2 (labeled C in Figure 4) and the non-proportional odds with ρ_0_ = 0.02 (labeled D) were both “steep” compared to the proportional odds model in the sense that the increase in the probability of toxicity occurred more rapidly over a smaller range of doses. The normal distribution with σ = 0.5 (labeled B) and the non-proportional odds with ρ_0_ = 0.126 (labeled E) were both “flat” compared to the proportional odds model because the increase in the probability of toxicity was slower and over a larger range of doses (see Figure 1 for a comparison).

The *EWOC* and *EWOC-PO* designs were routinely better in terms of efficiency than the AT designs when the true MTD was 0.5 or 0.7. The more detailed results from the proportional odds model, which can be seen in Figure 2, show that the *EWOC* and *EWOC-PO* designs had smaller absolute bias, smaller MSE, and higher proportions of estimated MTDs within an optimal range. When the true MTD was 0.1 the differences in efficiency between designs were difficult to distinguish.

The median for the average bias in the AT designs that were tested (Figure 4) was larger in absolute value than the medians for the *EWOC* or *EWOC-PO* designs for each of the response models. The inter-quartile range was also larger for the AT designs in each of the models. When looking across the different response models, the flat models in B and E tended to have more absolute bias and a greater range in average bias for all three study designs, while the steep models in C and D seemed to perform better.

Larger average root MSE occurred in the flat models B and E while the steep models in C and D had smaller ranges for the average root MSE (Figure 4). The largest average root MSE and the largest inter-quartile range for the average root MSE was found in the AT design for all models.

For all of models, *EWOC* and *EWOC-PO* designs had higher median average proportions of estimated MTDs within 15% of the true MTD compared to the AT designs (Figure 4). The first quartile in the *EWOC* and *EWOC-PO* designs was also higher than the third quartile in the AT designs. The steep models in C and D had almost uniformly higher average proportions than the flat models in B and E for all of the designs.

Safety in the *EWOC* designs was defined by setting the parameter theta. Both *EWOC* and *EWOC-PO* designs targeted that level and for the proportional odds model were safe in the sense that on average the proportion of DLTs did not exceed theta (the maximum for both was 0.34 in the proportional odds model). There is no corresponding parameter to consider when using MF-UD designs such as AT, although investigations into the operating characteristics of the traditional MF-UD design found a lower expected DLT rate at the MTD, from about 19%–22% with 13 dose levels and a logistic response function[25], to 18.9%–29% with 6 dose levels [20]. In our simulations of AT trials, the average DLT rate was 0.228. It can be seen in Figure 3 (Average proportion of DLTs) that the AT designs had outliers that were not safe. All of these outliers represent the trials where the true MTD was 0.1 and the starting dose of the AT design was also 0.1. The first dose was set to have no DLT, so the AT design algorithm always increased for the second dose, which caused these trials to always begin with a dose that was higher than the true MTD. Safety for the *EWOC* designs and the AT designs was also affected under the different models. There were scenarios where the *EWOC* and *EWOC-PO* designs exceeded the safety threshold under the flat models, but these *EWOC* designs were safe under the steep models.

The flat models in B and E had a higher median for the average proportion of DLTs in each of the designs (Figure 4). The *EWOC* designs had similar medians across models, although the flat models tended to be higher. The AT designs had the lowest medians across the models, but there were very large outliers for the AT designs that had a starting dose of 0.1 when the true MTD was 0.1 (three points for each ρ_1_ in each of B, C, D, E, and there were more AT designs run under proportional odds, so there are more data points outlying in A).

The proportion of trials with DLT rate above 0.38 (θ + 0.05) can be seen in Figure 4. The AT designs had large outliers when the starting dose and true MTD were 0.1, but in other scenarios the average proportions were low. The steep models in C and D showed the smallest ranges for these proportions in the *EWOC* and *EWOC-PO* designs. The proportion of patients receiving doses within an optimal range of the true MTD is compared under the different models in Figure 4. The *EWOC* and *EWOC-PO* designs had higher median average proportions than the AT designs and even the inter-quartile ranges did not overlap, except in B for *EWOC* and AT. Again, the steep models C and D tended to have higher proportions than the flat models in B and E.

Although the main goal of the simulation study was to compare the operating characteristics of the AT design with *EWOC* and *EWOC-PO*, there are slight differences in the results between the two EWOC designs. *EWOC-PO* estimates an additional parameter compared to *EWOC*, so the bias and root MSE can be slightly larger in scenarios where the probability of grade 2 responses are higher and affect dose escalation, but *EWOC-PO* is slightly safer when the probability of grade 2 responses are higher and the true MTD is low. These results confirm those previously reported by [14].

## 4. Concluding Remarks

Both *EWOC* and *EWOC-PO* designs tend to have smaller absolute bias than the AT designs, particularly when the true MTD is high. A higher proportion of trials with estimated MTD within an optimal range of the true MTD were obtained with the *EWOC* and *EWOC-PO* designs. These *EWOC* designs also tend to have lower MSE than the AT designs. The average proportion of DLTs for the AT designs were mostly between 0.1 and 0.25, although when the starting dose was equal to the true MTD the proportion was much higher. The *EWOC* and *EWOC-PO* designs targeted a DLT rate of 0.33 and the average proportion of DLTs in the simulations was usually a bit lower than 0.33, the exception being when the true MTD was low at 0.1. The proportion of patients receiving doses within 15% of true MTD (optimal doses) are uniformly higher with *EWOC* and *EWOC-PO* designs even when the model is misspecified. We conclude that EWOC designs are more efficient in estimating the MTD than AT designs and are safer under some scenarios. From the clinical investigator's perspective, lower bias and MSE make EWOC designs preferable than AT designs without compromising safety. From a patient's perspective, uniformly higher proportion of patients receiving doses within an optimal range of the true MTD makes EWOC designs preferable than AT designs.

A web portal [26] was established to facilitate access to software applications developed by our group to design and conduct dose finding clinical trials in cancer using the EWOC method: Standalone-EWOC application available for download, and Web-EWOC, a web-based calculator which includes documentation, storage and forum. Information about both the software and the method itself is available at that website to facilitate the implementation of EWOC in cancer trials. Also available at the same location is a tutorial which provides step-by-step instructions on how to design a trial using EWOC and use a simulation module that generates the operating characteristics of a particular design. The last section of the tutorial illustrates how all the information generated can be incorporated into a template to produce the statistical section of the dose finding protocol to be submitted to a Protocol Review Committee and Institutional Review Board.

We believe that although the FDA has the authority to demand the implementation of any design, cancer patients should not be exposed to designs shown to be sub-optimal. As suggested in [17]:

“… the most effective and fastest way to improve the present situation is a change in the attitude of the gatekeeper—regulatory agencies. The US Food and Drug Administration should proactively encourage the adoption of statistical designs that would allow more patients to be treated at near-optimal doses while controlling for excessive toxicity. After all, the real justification for using the standard modified Fibonacci up-and-down design is tradition (i.e., “This is how we always have done it”) and comfort level, not scientific reasoning or clinical evidence.”

What can be done to move towards the implementation of safer and more efficient designs for dose-finding clinical trials? If the designs demonstrated to be currently optimal are not even part of the discussion in a regulatory framework, the science in pharmaceutical companies and within FDA cannot advance. We hope that the present study will help to increase the dialog between research biostatisticians dedicated to the discovery and improvement of clinical trial design and the FDA in order to translate safer and more efficient designs into clinical trials and accelerate our progress against cancer.
